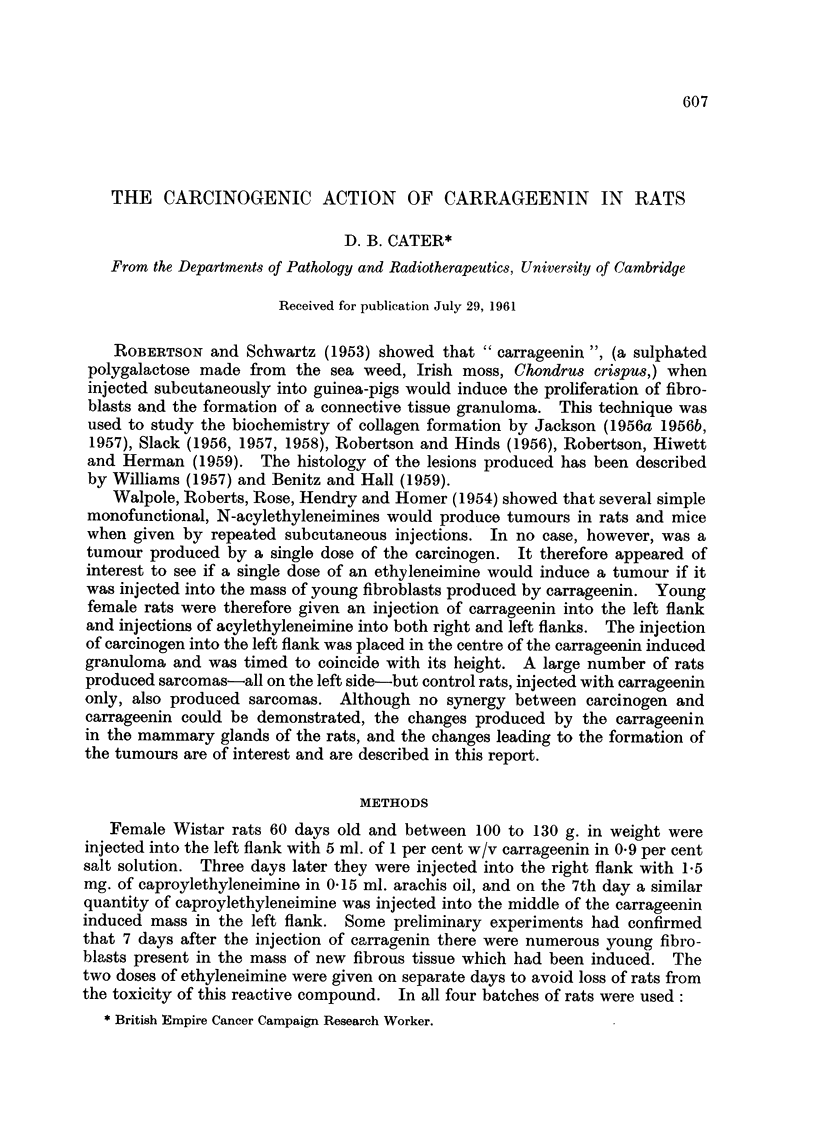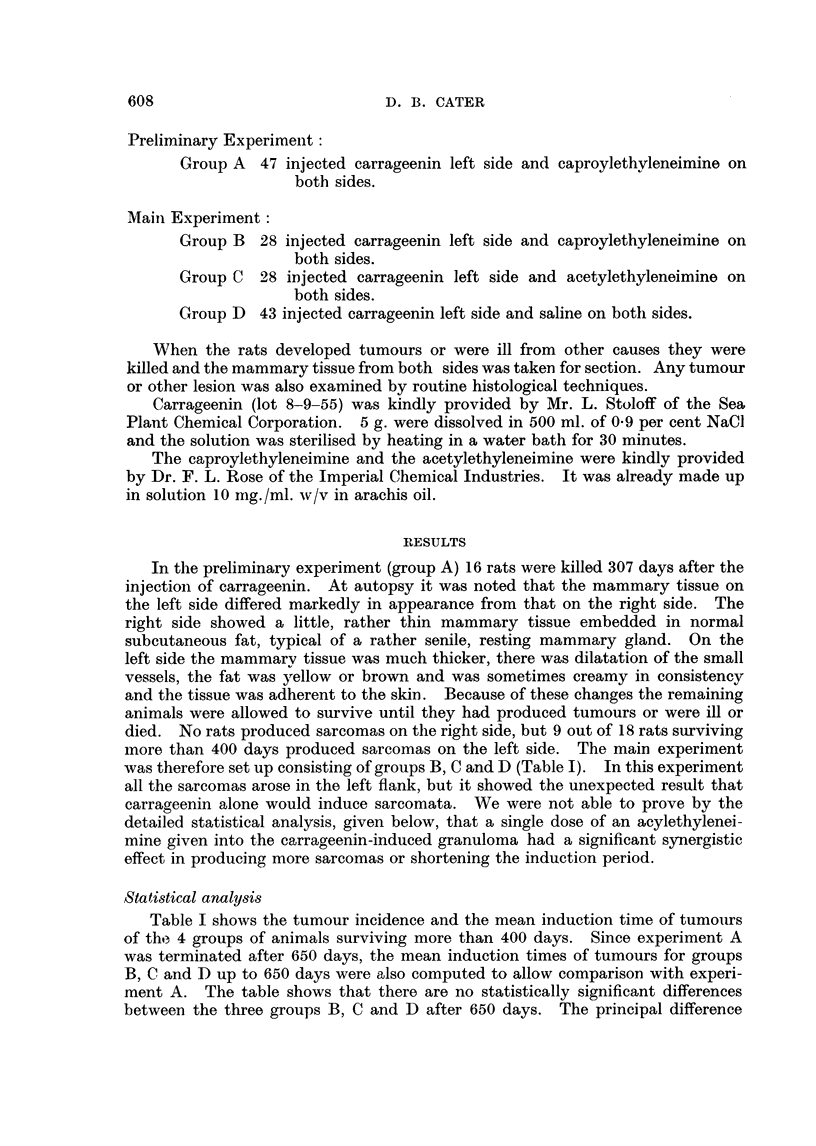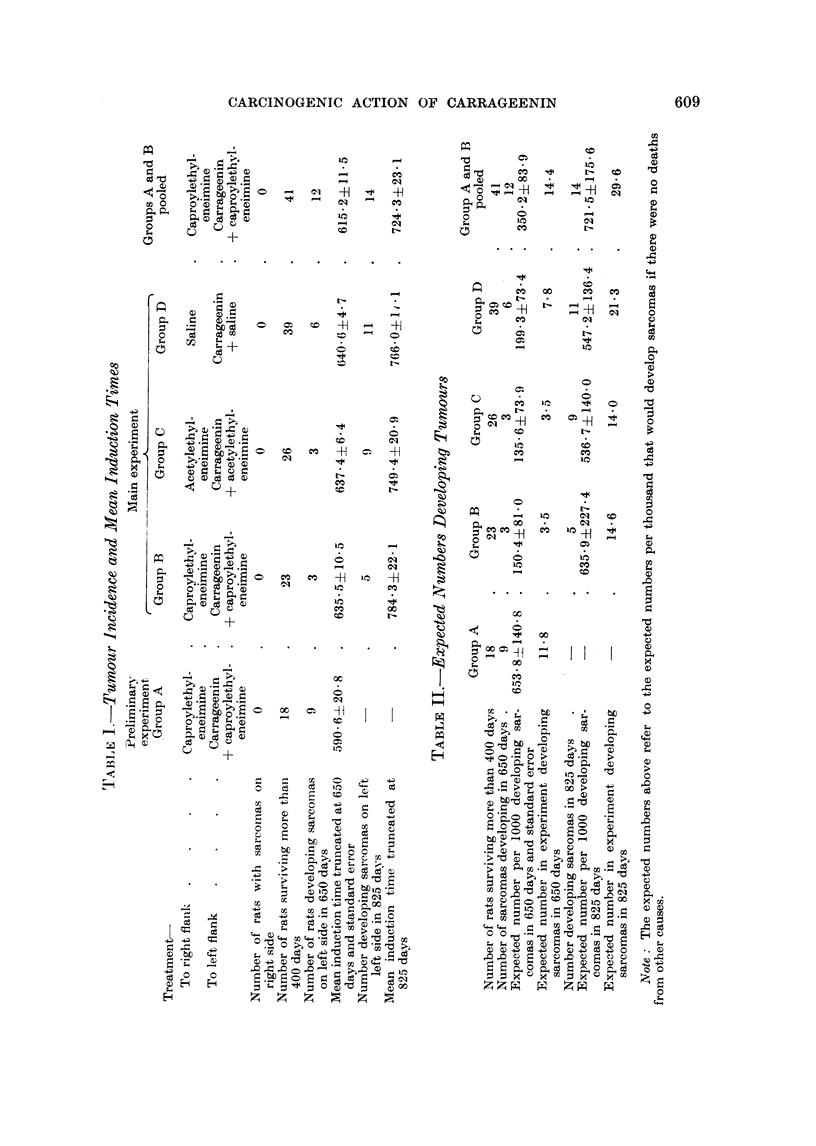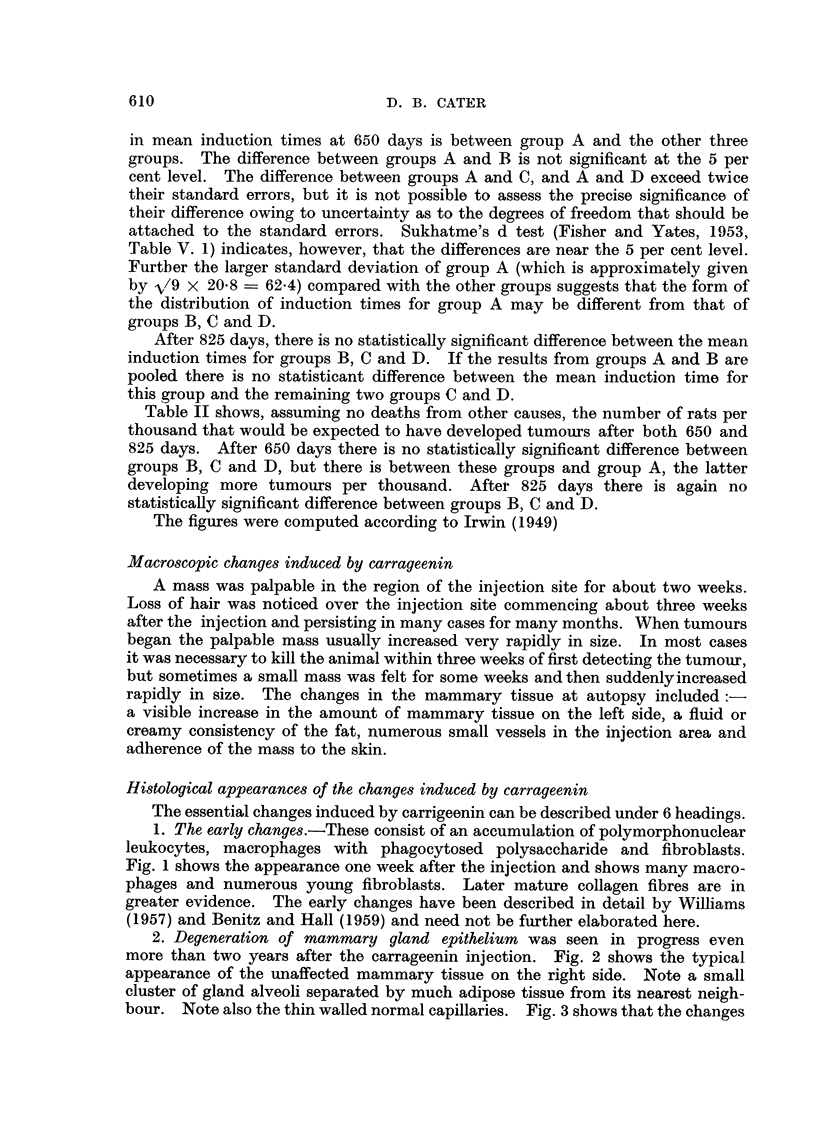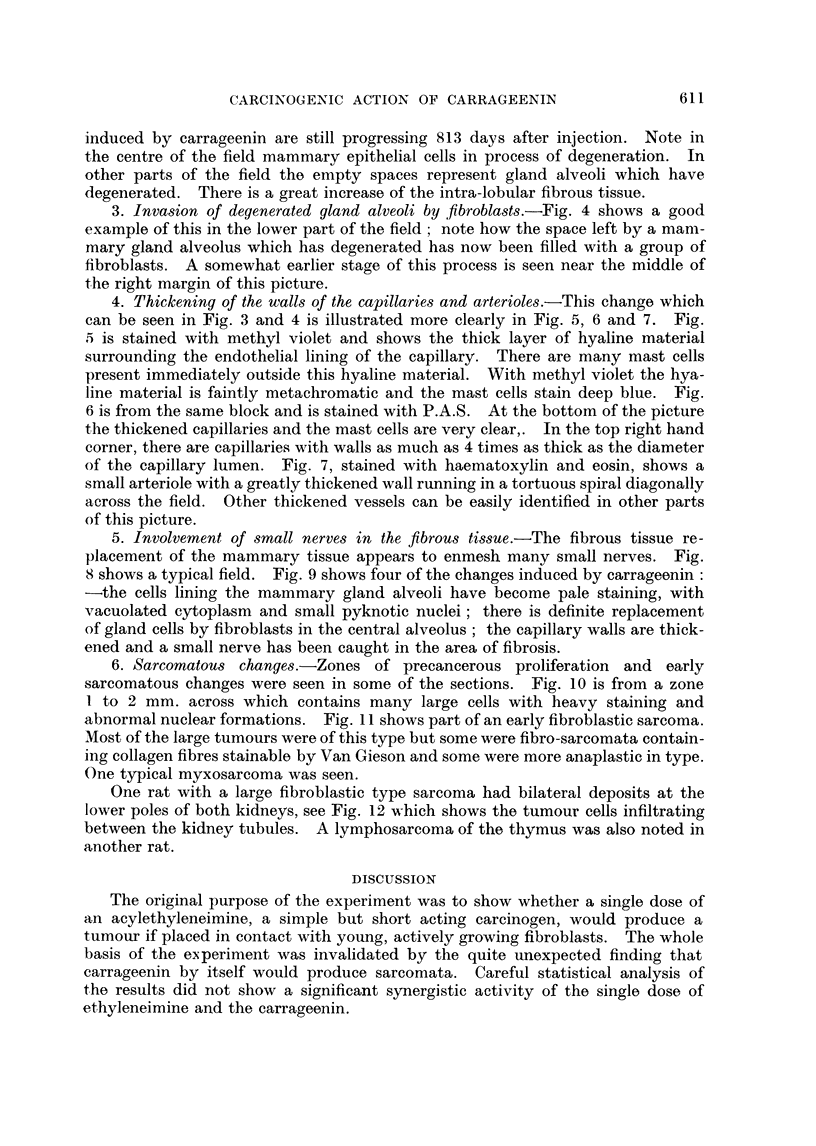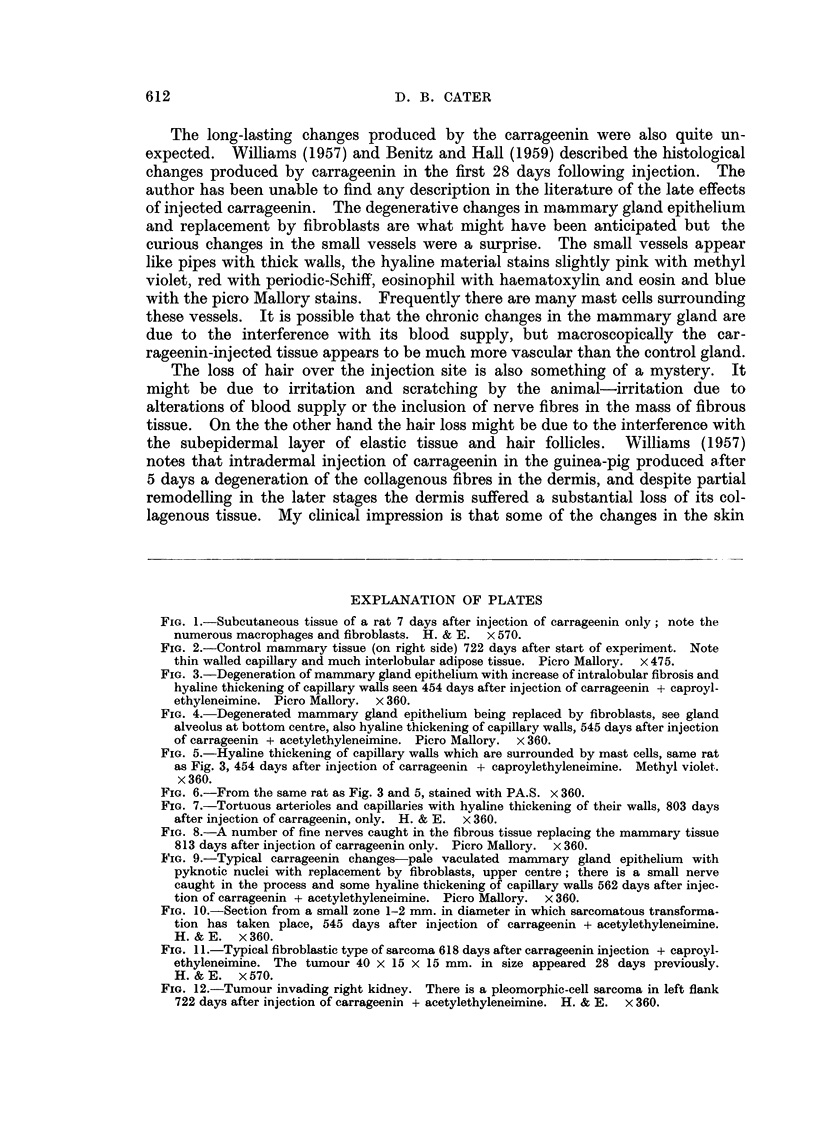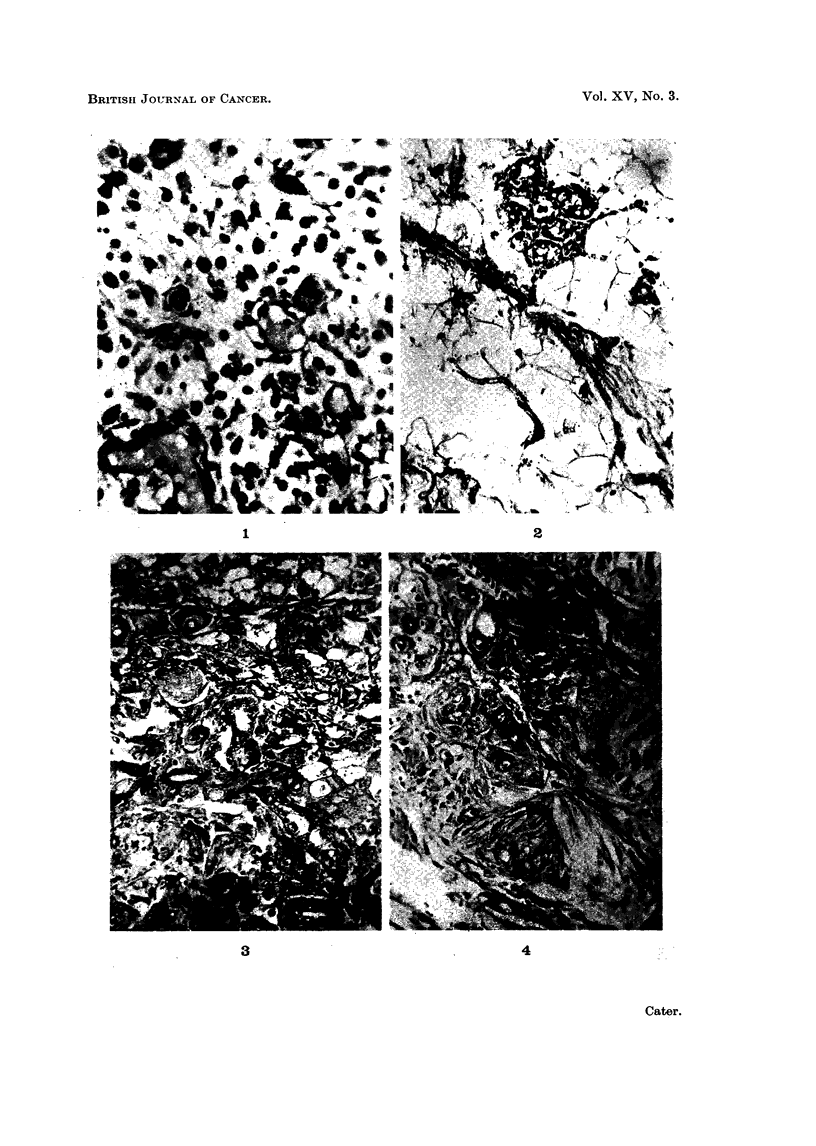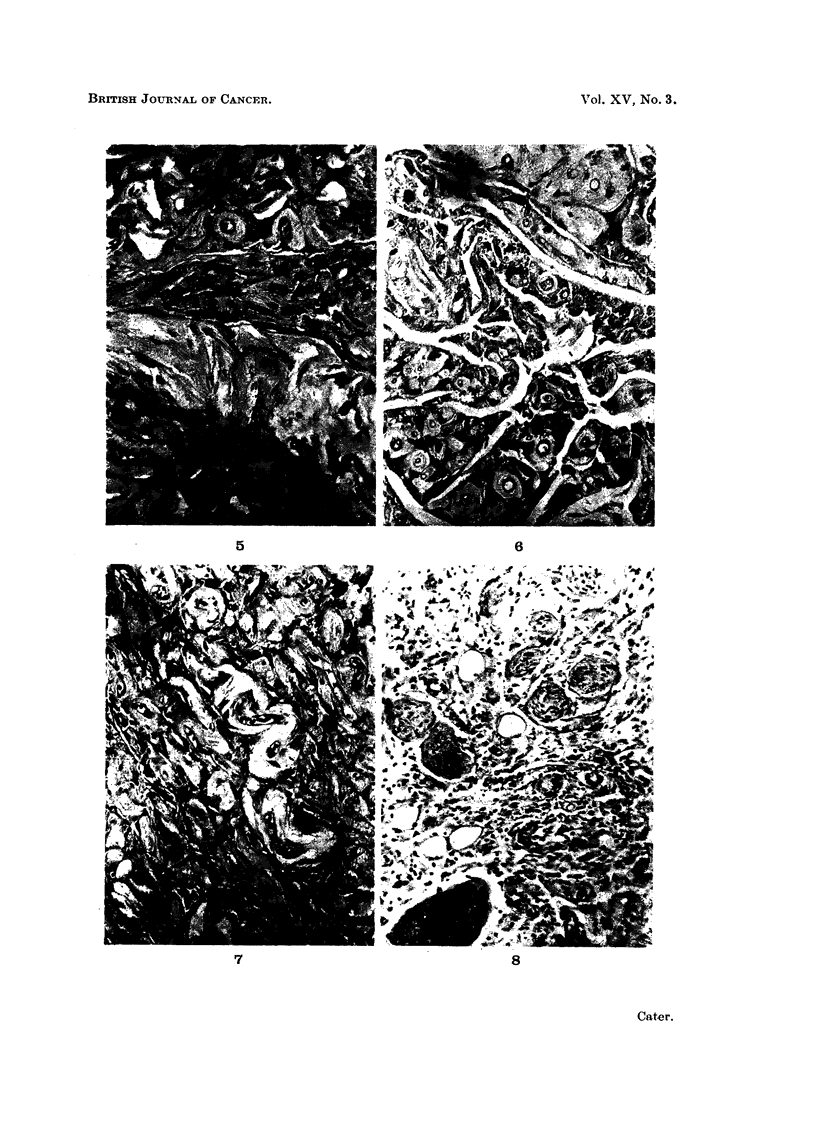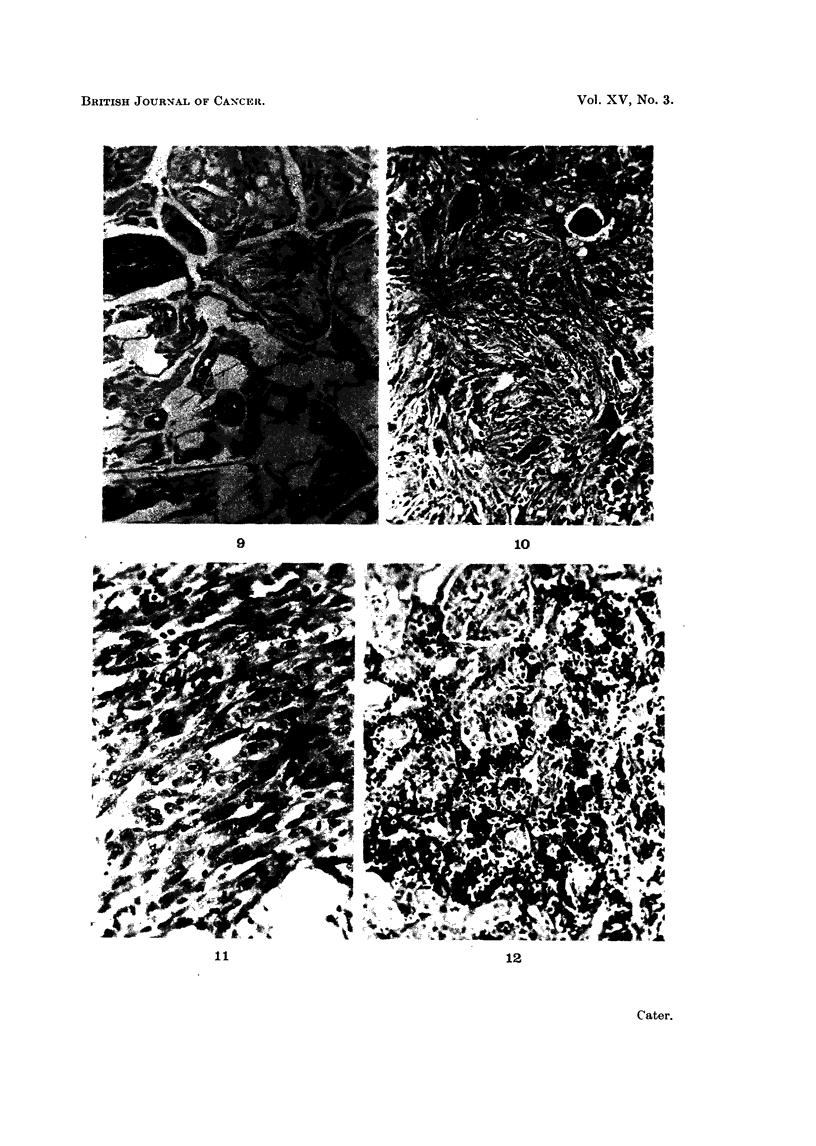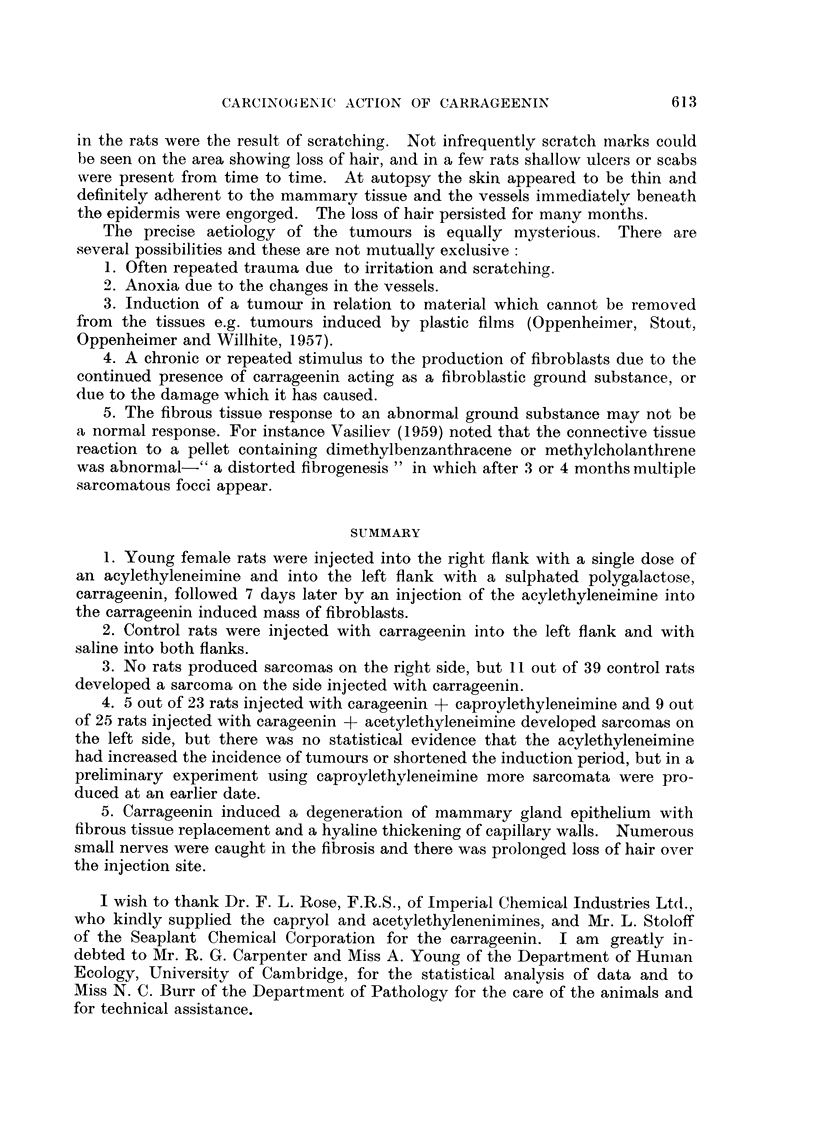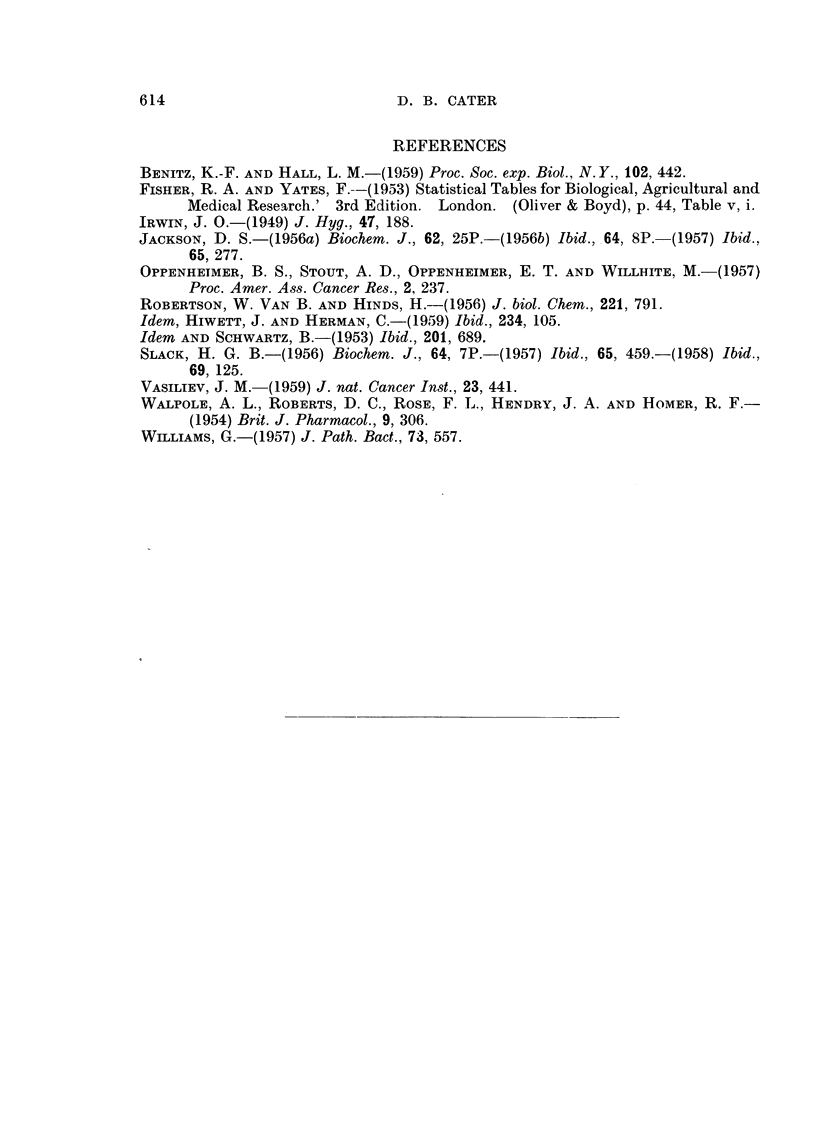# The Carcinogenic Action of Carrageenin in Rats

**DOI:** 10.1038/bjc.1961.70

**Published:** 1961-09

**Authors:** D. B. Cater

## Abstract

**Images:**


### THE CARCINOGENIC ACTION OF CARRAGEENIN IN RATS

D. B. CATER*

*From the Departments of Pathology and Radiotherapeutics, University of Cambridge*

Received for publication July 29, 1961

ROBERTSON and Schwartz (1953) showed that " carrageenin ", (a sulphated polygalactose made from the sea weed, Irish moss, *Chondrus crispus*,) when injected subcutaneously into guinea-pigs would induce the proliferation of fibroblasts and the formation of a connective tissue granuloma. This technique was used to study the biochemistry of collagen formation by Jackson (1956*a* 1956*b*, 1957), Slack (1956, 1957, 1958), Robertson and Hinds (1956), Robertson, Hiwett and Herman (1959). The histology of the lesions produced has been described by Williams (1957) and Benitz and Hall (1959).

Walpole, Roberts, Rose, Hendry and Homer (1954) showed that several simple monofunctional, N-acylethyleneimines would produce tumours in rats and mice when given by repeated subcutaneous injections. In no case, however, was a tumour produced by a single dose of the carcinogen. It therefore appeared of interest to see if a single dose of an ethyleneimine would induce a tumour if it was injected into the mass of young fibroblasts produced by carrageenin. Young female rats were therefore given an injection of carrageenin into the left flank and injections of acylethyleneimine into both right and left flanks. The injection of carcinogen into the left flank was placed in the centre of the carrageenin induced granuloma and was timed to coincide with its height. A large number of rats produced sarcomas—all on the left side—but control rats, injected with carrageenin only, also produced sarcomas. Although no synergy between carcinogen and carrageenin could be demonstrated, the changes produced by the carrageenin in the mammary glands of the rats, and the changes leading to the formation of the tumours are of interest and are described in this report.

### METHODS

Female Wistar rats 60 days old and between 100 to 130 g. in weight were injected into the left flank with 5 ml. of 1 per cent w/v carrageenin in 0·9 per cent salt solution. Three days later they were injected into the right flank with 1·5 mg. of caproylethyleneimine in 0·15 ml. arachis oil, and on the 7th day a similar quantity of caproylethyleneimine was injected into the middle of the carrageenin induced mass in the left flank. Some preliminary experiments had confirmed that 7 days after the injection of carragenin there were numerous young fibroblasts present in the mass of new fibrous tissue which had been induced. The two doses of ethyleneimine were given on separate days to avoid loss of rats from the toxicity of this reactive compound. In all four batches of rats were used :

* British Empire Cancer Campaign Research Worker.

Preliminary Experiment :

Group A 47 injected carrageenin left side and caproylethyleneimine on both sides.

Main Experiment :

Group B 28 injected carrageenin left side and caproylethyleneimine on both sides.

Group C 28 injected carrageenin left side and acetylethyleneimine on both sides.

Group D 43 injected carrageenin left side and saline on both sides.

When the rats developed tumours or were ill from other causes they were killed and the mammary tissue from both sides was taken for section. Any tumour or other lesion was also examined by routine histological techniques.

Carrageenin (lot 8–9–55) was kindly provided by Mr. L. Stoloff of the Sea Plant Chemical Corporation. 5 g. were dissolved in 500 ml. of 0·9 per cent NaCl and the solution was sterilised by heating in a water bath for 30 minutes.

The caproylethyleneimine and the acetylethyleneimine were kindly provided by Dr. F. L. Rose of the Imperial Chemical Industries. It was already made up in solution 10 mg./ml. w/v in arachis oil.

### RESULTS

In the preliminary experiment (group A) 16 rats were killed 307 days after the injection of carrageenin. At autopsy it was noted that the mammary tissue on the left side differed markedly in appearance from that on the right side. The right side showed a little, rather thin mammary tissue embedded in normal subcutaneous fat, typical of a rather senile, resting mammary gland. On the left side the mammary tissue was much thicker, there was dilatation of the small vessels, the fat was yellow or brown and was sometimes creamy in consistency and the tissue was adherent to the skin. Because of these changes the remaining animals were allowed to survive until they had produced tumours or were ill or died. No rats produced sarcomas on the right side, but 9 out of 18 rats surviving more than 400 days produced sarcomas on the left side. The main experiment was therefore set up consisting of groups B, C and D (Table I). In this experiment all the sarcomas arose in the left flank, but it showed the unexpected result that carrageenin alone would induce sarcomata. We were not able to prove by the detailed statistical analysis, given below, that a single dose of an acylethyleneimine given into the carrageenin-induced granuloma had a significant synergistic effect in producing more sarcomas or shortening the induction period.

### *Statistical analysis*

Table I shows the tumour incidence and the mean induction time of tumours of the 4 groups of animals surviving more than 400 days. Since experiment A was terminated after 650 days, the mean induction times of tumours for groups B, C and D up to 650 days were also computed to allow comparison with experiment A. The table shows that there are no statistically significant differences between the three groups B, C and D after 650 days. The principal difference

TABLE I.—*Tumour Incidence and Mean Induction Times*

| | Preliminary experiment Group A | Main experiment Group B | Main experiment Group C | Main experiment Group D | Groups A and B pooled |
|---|---|---|---|---|---|
| Treatment— | | | | | |
| To right flank | Caproylethyl-eneimine | Caproylethyl-eneimine | Acetylethyl-eneimine | Saline | Caproylethyl-eneimine |
| To left flank | Carrageenin + caproylethyl-eneimine | Carrageenin + caproylethyl-eneimine | Carrageenin + acetylethyl-eneimine | Carrageenin + saline | Carrageenin + caproylethyl-eneimine |
| Number of rats with sarcomas on right side | 0 | 0 | 0 | 0 | 0 |
| Number of rats surviving more than 400 days | 18 | 23 | 26 | 39 | 41 |
| Number of rats developing sarcomas on left side in 650 days | 9 | 3 | 3 | 6 | 12 |
| Mean induction time truncated at 650 days and standard error | 590·6±20·8 | 635·5±10·5 | 637·4±6·4 | 640·6±4·7 | 615·2±11·5 |
| Number developing sarcomas on left left side in 825 days | — | 5 | 9 | 11 | 14 |
| Mean induction time truncated at 825 days | — | 784·3±22·1 | 749·4±20·9 | 766·0±17·1 | 724·3±23·1 |

TABLE II.—*Expected Numbers Developing Tumours*

| | Group A | Group B | Group C | Group D | Group A and B pooled |
|---|---|---|---|---|---|
| Number of rats surviving more than 400 days | 18 | 23 | 26 | 39 | 41 |
| Number of sarcomas developing in 650 days | 9 | 3 | 3 | 6 | 12 |
| Expected number per 1000 developing sarcomas in 650 days and standard error | 653·8±140·8 | 150·4±81·0 | 135·6±73·9 | 199·3±73·4 | 350·2±83·9 |
| Expected number in experiment developing sarcomas in 650 days | 11·8 | 3·5 | 3·5 | 7·8 | 14·4 |
| Number developing sarcomas in 825 days | — | 5 | 9 | 11 | 14 |
| Expected number per 1000 developing sarcomas in 825 days | — | 635·9±227·4 | 536·7±140·0 | 547·2±136·4 | 721·5±175·6 |
| Expected number in experiment developing sarcomas in 825 days | — | 14·6 | 14·0 | 21·3 | 29·6 |

*Note :* The expected numbers above refer to the expected numbers per thousand that would develop sarcomas if there were no deaths from other causes.

in mean induction times at 650 days is between group A and the other three groups. The difference between groups A and B is not significant at the 5 per cent level. The difference between groups A and C, and A and D exceed twice their standard errors, but it is not possible to assess the precise significance of their difference owing to uncertainty as to the degrees of freedom that should be attached to the standard errors. Sukhatme's d test (Fisher and Yates, 1953, Table V. 1) indicates, however, that the differences are near the 5 per cent level. Further the larger standard deviation of group A (which is approximately given by $\sqrt{9} \times 20{\cdot}8 = 62{\cdot}4$) compared with the other groups suggests that the form of the distribution of induction times for group A may be different from that of groups B, C and D.

After 825 days, there is no statistically significant difference between the mean induction times for groups B, C and D. If the results from groups A and B are pooled there is no statisticant difference between the mean induction time for this group and the remaining two groups C and D.

Table II shows, assuming no deaths from other causes, the number of rats per thousand that would be expected to have developed tumours after both 650 and 825 days. After 650 days there is no statistically significant difference between groups B, C and D, but there is between these groups and group A, the latter developing more tumours per thousand. After 825 days there is again no statistically significant difference between groups B, C and D.

The figures were computed according to Irwin (1949)

*Macroscopic changes induced by carrageenin*

A mass was palpable in the region of the injection site for about two weeks. Loss of hair was noticed over the injection site commencing about three weeks after the injection and persisting in many cases for many months. When tumours began the palpable mass usually increased very rapidly in size. In most cases it was necessary to kill the animal within three weeks of first detecting the tumour, but sometimes a small mass was felt for some weeks and then suddenly increased rapidly in size. The changes in the mammary tissue at autopsy included :— a visible increase in the amount of mammary tissue on the left side, a fluid or creamy consistency of the fat, numerous small vessels in the injection area and adherence of the mass to the skin.

*Histological appearances of the changes induced by carrageenin*

The essential changes induced by carrigeenin can be described under 6 headings.

1. *The early changes.*—These consist of an accumulation of polymorphonuclear leukocytes, macrophages with phagocytosed polysaccharide and fibroblasts. Fig. 1 shows the appearance one week after the injection and shows many macrophages and numerous young fibroblasts. Later mature collagen fibres are in greater evidence. The early changes have been described in detail by Williams (1957) and Benitz and Hall (1959) and need not be further elaborated here.

2. *Degeneration of mammary gland epithelium* was seen in progress even more than two years after the carrageenin injection. Fig. 2 shows the typical appearance of the unaffected mammary tissue on the right side. Note a small cluster of gland alveoli separated by much adipose tissue from its nearest neighbour. Note also the thin walled normal capillaries. Fig. 3 shows that the changes induced by carrageenin are still progressing 813 days after injection. Note in the centre of the field mammary epithelial cells in process of degeneration. In other parts of the field the empty spaces represent gland alveoli which have degenerated. There is a great increase of the intra-lobular fibrous tissue.

3. *Invasion of degenerated gland alveoli by fibroblasts.*—Fig. 4 shows a good example of this in the lower part of the field ; note how the space left by a mammary gland alveolus which has degenerated has now been filled with a group of fibroblasts. A somewhat earlier stage of this process is seen near the middle of the right margin of this picture.

4. *Thickening of the walls of the capillaries and arterioles.*—This change which can be seen in Fig. 3 and 4 is illustrated more clearly in Fig. 5, 6 and 7. Fig. 5 is stained with methyl violet and shows the thick layer of hyaline material surrounding the endothelial lining of the capillary. There are many mast cells present immediately outside this hyaline material. With methyl violet the hyaline material is faintly metachromatic and the mast cells stain deep blue. Fig. 6 is from the same block and is stained with P.A.S. At the bottom of the picture the thickened capillaries and the mast cells are very clear,. In the top right hand corner, there are capillaries with walls as much as 4 times as thick as the diameter of the capillary lumen. Fig. 7, stained with haematoxylin and eosin, shows a small arteriole with a greatly thickened wall running in a tortuous spiral diagonally across the field. Other thickened vessels can be easily identified in other parts of this picture.

5. *Involvement of small nerves in the fibrous tissue.*—The fibrous tissue replacement of the mammary tissue appears to enmesh many small nerves. Fig. 8 shows a typical field. Fig. 9 shows four of the changes induced by carrageenin : —the cells lining the mammary gland alveoli have become pale staining, with vacuolated cytoplasm and small pyknotic nuclei ; there is definite replacement of gland cells by fibroblasts in the central alveolus ; the capillary walls are thickened and a small nerve has been caught in the area of fibrosis.

6. *Sarcomatous changes.*—Zones of precancerous proliferation and early sarcomatous changes were seen in some of the sections. Fig. 10 is from a zone 1 to 2 mm. across which contains many large cells with heavy staining and abnormal nuclear formations. Fig. 11 shows part of an early fibroblastic sarcoma. Most of the large tumours were of this type but some were fibro-sarcomata containing collagen fibres stainable by Van Gieson and some were more anaplastic in type. One typical myxosarcoma was seen.

One rat with a large fibroblastic type sarcoma had bilateral deposits at the lower poles of both kidneys, see Fig. 12 which shows the tumour cells infiltrating between the kidney tubules. A lymphosarcoma of the thymus was also noted in another rat.

### DISCUSSION

The original purpose of the experiment was to show whether a single dose of an acylethyleneimine, a simple but short acting carcinogen, would produce a tumour if placed in contact with young, actively growing fibroblasts. The whole basis of the experiment was invalidated by the quite unexpected finding that carrageenin by itself would produce sarcomata. Careful statistical analysis of the results did not show a significant synergistic activity of the single dose of ethyleneimine and the carrageenin.

The long-lasting changes produced by the carrageenin were also quite unexpected. Williams (1957) and Benitz and Hall (1959) described the histological changes produced by carrageenin in the first 28 days following injection. The author has been unable to find any description in the literature of the late effects of injected carrageenin. The degenerative changes in mammary gland epithelium and replacement by fibroblasts are what might have been anticipated but the curious changes in the small vessels were a surprise. The small vessels appear like pipes with thick walls, the hyaline material stains slightly pink with methyl violet, red with periodic-Schiff, eosinophil with haematoxylin and eosin and blue with the picro Mallory stains. Frequently there are many mast cells surrounding these vessels. It is possible that the chronic changes in the mammary gland are due to the interference with its blood supply, but macroscopically the carrageenin-injected tissue appears to be much more vascular than the control gland.

The loss of hair over the injection site is also something of a mystery. It might be due to irritation and scratching by the animal—irritation due to alterations of blood supply or the inclusion of nerve fibres in the mass of fibrous tissue. On the the other hand the hair loss might be due to the interference with the subepidermal layer of elastic tissue and hair follicles. Williams (1957) notes that intradermal injection of carrageenin in the guinea-pig produced after 5 days a degeneration of the collagenous fibres in the dermis, and despite partial remodelling in the later stages the dermis suffered a substantial loss of its collagenous tissue. My clinical impression is that some of the changes in the skin

---

### EXPLANATION OF PLATES

Fig. 1.—Subcutaneous tissue of a rat 7 days after injection of carrageenin only; note the numerous macrophages and fibroblasts. H. & E. ×570.

Fig. 2.—Control mammary tissue (on right side) 722 days after start of experiment. Note thin walled capillary and much interlobular adipose tissue. Picro Mallory. ×475.

Fig. 3.—Degeneration of mammary gland epithelium with increase of intralobular fibrosis and hyaline thickening of capillary walls seen 454 days after injection of carrageenin + caproylethyleneimine. Picro Mallory. ×360.

Fig. 4.—Degenerated mammary gland epithelium being replaced by fibroblasts, see gland alveolus at bottom centre, also hyaline thickening of capillary walls, 545 days after injection of carrageenin + acetylethyleneimine. Picro Mallory. ×360.

Fig. 5.—Hyaline thickening of capillary walls which are surrounded by mast cells, same rat as Fig. 3, 454 days after injection of carrageenin + caproylethyleneimine. Methyl violet. ×360.

Fig. 6.—From the same rat as Fig. 3 and 5, stained with PA.S. ×360.

Fig. 7.—Tortuous arterioles and capillaries with hyaline thickening of their walls, 803 days after injection of carrageenin, only. H. & E. ×360.

Fig. 8.—A number of fine nerves caught in the fibrous tissue replacing the mammary tissue 813 days after injection of carrageenin only. Picro Mallory. ×360.

Fig. 9.—Typical carrageenin changes—pale vaculated mammary gland epithelium with pyknotic nuclei with replacement by fibroblasts, upper centre; there is a small nerve caught in the process and some hyaline thickening of capillary walls 562 days after injection of carrageenin + acetylethyleneimine. Picro Mallory. ×360.

Fig. 10.—Section from a small zone 1–2 mm. in diameter in which sarcomatous transformation has taken place, 545 days after injection of carrageenin + acetylethyleneimine. H. & E. ×360.

Fig. 11.—Typical fibroblastic type of sarcoma 618 days after carrageenin injection + caproylethyleneimine. The tumour 40 × 15 × 15 mm. in size appeared 28 days previously. H. & E. ×570.

Fig. 12.—Tumour invading right kidney. There is a pleomorphic-cell sarcoma in left flank 722 days after injection of carrageenin + acetylethyleneimine. H. & E. ×360.

1

2

3

4

5

6

7

8

9

10

11

12

Cater.

in the rats were the result of scratching. Not infrequently scratch marks could be seen on the area showing loss of hair, and in a few rats shallow ulcers or scabs were present from time to time. At autopsy the skin appeared to be thin and definitely adherent to the mammary tissue and the vessels immediately beneath the epidermis were engorged. The loss of hair persisted for many months.

The precise aetiology of the tumours is equally mysterious. There are several possibilities and these are not mutually exclusive :

1. Often repeated trauma due to irritation and scratching.
2. Anoxia due to the changes in the vessels.
3. Induction of a tumour in relation to material which cannot be removed from the tissues e.g. tumours induced by plastic films (Oppenheimer, Stout, Oppenheimer and Willhite, 1957).
4. A chronic or repeated stimulus to the production of fibroblasts due to the continued presence of carrageenin acting as a fibroblastic ground substance, or due to the damage which it has caused.
5. The fibrous tissue response to an abnormal ground substance may not be a normal response. For instance Vasiliev (1959) noted that the connective tissue reaction to a pellet containing dimethylbenzanthracene or methylcholanthrene was abnormal—" a distorted fibrogenesis " in which after 3 or 4 months multiple sarcomatous focci appear.

### SUMMARY

1. Young female rats were injected into the right flank with a single dose of an acylethyleneimine and into the left flank with a sulphated polygalactose, carrageenin, followed 7 days later by an injection of the acylethyleneimine into the carrageenin induced mass of fibroblasts.
2. Control rats were injected with carrageenin into the left flank and with saline into both flanks.
3. No rats produced sarcomas on the right side, but 11 out of 39 control rats developed a sarcoma on the side injected with carrageenin.
4. 5 out of 23 rats injected with carageenin + caproylethyleneimine and 9 out of 25 rats injected with carageenin + acetylethyleneimine developed sarcomas on the left side, but there was no statistical evidence that the acylethyleneimine had increased the incidence of tumours or shortened the induction period, but in a preliminary experiment using caproylethyleneimine more sarcomata were produced at an earlier date.
5. Carrageenin induced a degeneration of mammary gland epithelium with fibrous tissue replacement and a hyaline thickening of capillary walls. Numerous small nerves were caught in the fibrosis and there was prolonged loss of hair over the injection site.

I wish to thank Dr. F. L. Rose, F.R.S., of Imperial Chemical Industries Ltd., who kindly supplied the capryol and acetylethylenenimines, and Mr. L. Stoloff of the Seaplant Chemical Corporation for the carrageenin. I am greatly indebted to Mr. R. G. Carpenter and Miss A. Young of the Department of Human Ecology, University of Cambridge, for the statistical analysis of data and to Miss N. C. Burr of the Department of Pathology for the care of the animals and for technical assistance.

### REFERENCES

BENITZ, K.-F. AND HALL, L. M.—(1959) *Proc. Soc. exp. Biol., N.Y.*, **102**, 442.
FISHER, R. A. AND YATES, F.—(1953) Statistical Tables for Biological, Agricultural and Medical Research.' 3rd Edition. London. (Oliver & Boyd), p. 44, Table v, i.
IRWIN, J. O.—(1949) *J. Hyg.*, **47**, 188.
JACKSON, D. S.—(1956*a*) *Biochem. J.*, **62**, 25P.—(1956*b*) *Ibid.*, **64**, 8P.—(1957) *Ibid.*, **65**, 277.
OPPENHEIMER, B. S., STOUT, A. D., OPPENHEIMER, E. T. AND WILLHITE, M.—(1957) *Proc. Amer. Ass. Cancer Res.*, **2**, 237.
ROBERTSON, W. VAN B. AND HINDS, H.—(1956) *J. biol. Chem.*, **221**, 791.
*Idem*, HIWETT, J. AND HERMAN, C.—(1959) *Ibid.*, **234**, 105.
*Idem* AND SCHWARTZ, B.—(1953) *Ibid.*, **201**, 689.
SLACK, H. G. B.—(1956) *Biochem. J.*, **64**, 7P.—(1957) *Ibid.*, **65**, 459.—(1958) *Ibid.*, **69**, 125.
VASILIEV, J. M.—(1959) *J. nat. Cancer Inst.*, **23**, 441.
WALPOLE, A. L., ROBERTS, D. C., ROSE, F. L., HENDRY, J. A. AND HOMER, R. F.—(1954) *Brit. J. Pharmacol.*, **9**, 306.
WILLIAMS, G.—(1957) *J. Path. Bact.*, **73**, 557.